# Developing clinical practice guidelines for caries prevention and management for pre-school children through the ADAPTE process and Delphi consensus

**DOI:** 10.1186/s12961-016-0117-0

**Published:** 2016-06-14

**Authors:** Gillian H. M. Lee, Colman McGrath, Cynthia K. Y. Yiu

**Affiliations:** Paediatric Dentistry, Faculty of Dentistry, University of Hong Kong, 2/F, Prince Philip Dental Hospital, 34 Hospital Road, Hong Kong, China; Periodontology and Public Health, Faculty of Dentistry, University of Hong Kong, 3/F, Prince Philip Dental Hospital, 34 Hospital Road, Hong Kong, China

**Keywords:** Guidelines, Guidelines development, ADAPTE, Delphi consensus, Oral health, Children, Caries risk assessment, Dental caries, Prevention

## Abstract

**Background:**

This study aims to develop consensus evidence-based clinical guidelines for caries prevention and management by caries risk assessment for pre-school children in Hong Kong.

**Methods:**

Employing the ADAPTE process, guidelines for caries prevention and management by caries risk assessment for pre-school children with a preliminary list of 91 recommendations was complied. External review of the guidelines was conducted by a panel of 41 reviewers from the *Hong Kong Society of Paediatric Dentistry* using a two-round web-based Delphi process. The reviewers were invited to contribute any comments on the draft-adapted guidelines and rated their agreement with each recommendation using a 9-point Likert scale. During the second round, 36 participants received anonymous feedback from the first round and assessed a narrowed list of 28 recommendations. Recommendations were retained and classified according to the median score and rating percentages by the reviewers.

**Results:**

A total of 70 out of 91 recommendations were retained (five reached high consensus, 65 reached consensus), and 21 recommendations were discarded. Recommendations and guidelines were outlined.

**Conclusions:**

Caries prevention and management guidelines for pre-school children were developed for use in Hong Kong using the ADAPTE process and Delphi consensus to develop evidence-based recommendations. This can facilitate the translation of guidelines into dental practice.

## Background

Dental caries in pre-school children remains a problem in Hong Kong. In a recent population-based oral health survey, the caries experience of pre-school children was over 50% in Hong Kong, with an extensive proportion of untreated carious teeth (over 90%) [1]. One in 20 of the children had a dental abscess associated with an extensively decayed tooth. The prevalence of dental caries among pre-school children showed limited improvement over the past decade and the extent and severity have increased. Among pre-school children, the number of decayed, missing and filled teeth was 2.3 in 2001 and 2.5 in 2011 [1, 2]. Children from disadvantaged and socially marginalised populations have even higher caries experience and are at higher risk for caries.

The high degree of untreated dental caries indicates that there is inadequate access to quality oral healthcare for pre-school children in Hong Kong. Management of pre-school children’s oral health requires commitment from professionals, patients and their families. Dentists’ attitudes can contribute to the problem, resulting in a barrier to provide adequate and quality dental care [3]. Dentists in Hong Kong do see the value of dental treatment for pre-school children, but most general dental practitioners consider children’s coping skills to dental treatment as a problem and have a negative attitude towards the provision of dental care for children [4]. A wide variation in caries management approaches among dental practitioners has also been identified [5], reflecting the uncertainty within the profession about the most suitable and effective dental care approaches for pre-school children with dental caries. Thus, these factors highlight the need for clinical guidelines relating to dental caries for pre-school children in Hong Kong.

Clinical practice guidelines are systematically developed statements that can assist practitioners and patients in making decisions about appropriate healthcare for specific clinical circumstances [6, 7]. It is the major available tool to assist in the translating of research evidence into practice. With the best available evidence and opinion in guidelines, healthcare professionals can be assisted in making appropriate clinical decisions, minimising variation in practice, and promote effective and safe patient outcomes.

Expectations and requirements have been proposed internationally for the development of quality clinical guidelines [8, 9]. The development and implementation of guidelines that meet international standards require substantial time, expertise and resources, especially during systematic identification and critical analysis of evidence. Guideline adaptation is a systematic approach for considering the endorsement or modification of guidelines produced in one setting for application and implementation in another as an alternative to de novo guideline development or as a first step in the process of implementation, while preserving evidence-based principles [10]. The ADAPTE process is a comprehensive framework for guideline adaptation. ADAPTE can reduce duplication of effort and enhance efficiency in guideline development. The manual and toolkit for the ADAPTE process are available at: http://www.g-i-n.net/gin [11]. The ADAPTE process consists of three main phases: set up, adaptation and finalisation.

The Delphi technique is a formal iterative structured process that aims to gather consensus of opinion, judgement or choice among a panel of experts [12–15]. The attitudes, needs and priorities of the panel can be explored during the interactive process. Delphi is suitable for reaching consensus on topics with dissenting opinions, uncertainty or absence of evidence. Guidelines developed with consensus of stakeholders are more likely to be accepted by the dental profession.

There are several quality guidelines for managing dental caries for pre-school children worldwide. Nevertheless, guidelines ascribed to other countries or areas can encounter barriers to their practical application in a context with a different culture and healthcare system [16]. Localised or adapted clinical guidelines on the basis of available evidence in the local context are advocated, with the consideration of cost-effectiveness and suitability for the local population. This project aims to develop consensus evidence-based clinical guidelines to inform the practice regarding the determination of caries risk and developing preventive and management strategies for pre-school children in Hong Kong using the ADAPTE process and a web-based Delphi consensus technique.

## Methods

### Set up phase

The project was led by a team of researchers based at the Faculty of Dentistry, the University of Hong Kong. The team comprised of specialists in the field of Dental Public Health and Paediatric Dentistry. Members of the team reported no conflict of interests. The scope and key questions for the guidelines were established. The key stakeholder, the *Hong Kong Society of Paediatric Dentistry* (HKSPD), was identified and invited to be the collaborator on developing the guidelines. An introduction to guideline development and the ADAPTE process was presented to the members of the Society.

### Adaptation phase

#### Search and screen guidelines

A search for relevant guidelines, protocols or policy statements in guideline repository websites, websites of various key guideline development organisations, and dental professional organisations, plus MEDLINE (Ovid), PubMed, Google and several other databases was conducted. The subject heading ‘dental caries’ was used for the search in combination with several other keyword terms: ‘tooth decay’ or ‘diagnosis’ or ‘management’ or ‘prevention’ or ‘approach’ or ‘caries risk’ or ‘caries risk assessment’ or ‘caries assessment’ or ‘caries prediction’ or ‘children’. An appropriate search strategy for each database was employed.

Twenty non-duplicate guidelines, protocols or policy statements were identified in the search. No language restrictions were applied at this point. The retrieved guidelines were screened according to list of a priori inclusion/exclusion criteria: (1) dental caries, regarding its diagnosis, prevention, management or risk assessment was the main topic of the guidelines; (2) the target population of the guidelines was pre-school children with primary dentition; (3) the guidelines were for local or national use; (4) publication date should be from 2000 onwards to date of the search (December 2011); (5) indication that a literature search, ideally using a systematic method, had been conducted; (6) references were included; and (7) explicit link between the recommendations and the supporting evidence was indicated.

#### Access guidelines

Eleven guidelines/protocols were retained for further assessment after initial screening based on the inclusion/exclusion criteria. A panel group with four clinical teaching staff from the Department of Paediatric Dentistry of the University of Hong Kong was set up. The panel members were briefed about the guideline development and evaluation process for the retrieved guidelines by the guideline development group. They were invited to review the 11 retrieved guidelines and appraise their quality using the AGREE II (Appraisal of Guidelines for Research and Evaluation II) instrument individually [17]. The AGREE II instrument is a generic instrument to assess the process of guideline development and reporting of this process in the guidelines. The instrument consists of 23 items on a 7-point response scale comprising six quality-related domains: scope and purpose, stakeholder involvement, rigour of development, clarity of presentation, applicability, and editorial independence. The instrument’s overall assessment includes the rating of the overall quality of the guideline and whether the guideline would be recommended for use in practice. The scores for each of the six AGREE II domains of each of the retrieved guidelines were calculated according to the rubrics. Pooled domain scores and overall assessment scores for the 11 guidelines are presented in Table 1.

**Table 1:** The six AGREE II domain scores and overall assessment scores of the 11 retrieved guidelines

Retrieved guidelines	Scaled domain score						
	Domain 1	Domain 2	Domain 3	Domain 4	Domain 5	Domain 6	Overall
Scottish Intercollegiate Guidelines Network, 2005 [18]	72.22	69.44	59.38	63.89	61.46	54.17	66.67
American Dental Association Council on Scientific Affairs, 2006 [33]	63.89	56.94	53.13	68.06	47.92	64.58	58.33
Ramos-Gomez et al., 2007 [34]	72.22	58.33	43.23	61.11	50.00	35.42	54.17
Irish Oral Health Services Guideline Initiative, 2008 [22]	93.06	77.78	80.73	81.94	67.71	64.58	83.33
Swedish Council on Technology Assessment in Health Care (SBU), 2008 [35]	65.28	51.39	53.13	56.94	50.00	37.50	54.17
American Academy of Pediatric Dentistry, 2009 [36]	59.72	36.11	30.73	47.22	36.46	43.75	45.83
Irish Oral Health Services Guideline Initiative, 2009 [19]	91.67	65.28	79.69	79.17	63.54	72.92	79.17
Scottish Dental Effectiveness Programme, 2010 [21]	76.39	69.44	60.94	72.22	65.63	66.67	70.83
Ramos-Gomez et al., 2010 [37]	68.06	55.56	47.40	59.72	54.17	43.75	54.17
American Academy of Pediatric Dentistry, 2011 [20]	76.39	66.67	56.77	66.67	52.08	41.67	62.50
Ramos-Gomez and Ng, 2011 [23]	72.22	63.89	58.33	62.50	51.04	39.58	54.17

Domain 1 – Scope & Purpose; Domain 2 – Stakeholder Involvement; Domain 3 – Rigour of Development; Domain 4 – Clarity of Presentation; Domain 5 – Applicability; Domain 6 – Editorial Independence; Overall – Overall assessment

Three ‘Source’ guidelines for adaptation in the ADAPTE process were selected [18–20]. The selection was based on the AGREE II scores on Domain 3 (rigour of development) and overall assessment scores. The currency of the guidelines, applicability and extent of the key questions for the guidelines were also taken into account. Although the Scottish Dental Effectiveness Programme 2010 [21] had higher scores than American Academy of Pediatric Dentistry 2011 [20], the guideline was not selected because the content and extent of key questions covered was very similar to the Scottish Intercollegiate Guidelines Network (SIGN) 2005 [18]. Therefore, the next guideline on the list, that of American Academy of Pediatric Dentistry 2011 [20], was selected. The three ‘source’ guidelines provided different perspectives on caries prevention and management strategies and on caries risk assessment, and together addressed most of the key questions for the developing guidelines. With regard to the clarity of presentation of the guidelines, the other three retrieved guidelines [21–23] were kept as ‘satellite’ for reference.

#### Drafting of guidelines

The recommendations from the three source guidelines were compiled in a recommendation matrix and compared. The grading and levels of evidence associated with each of the recommendations provided by the source guidelines were placed within each cell and was critically appraised using the SIGN methodology checklist [24].

The consistency between the evidence presented in the source guidelines, their interpretation of the evidence, and the resulting recommendations were evaluated. The original evidence supporting the interpretations and recommendations in the source guidelines were reviewed. The currency of the source guidelines for the adaptation process was also assessed to ascertain whether the most current evidence had been included.

A literature search of databases and websites for systematic reviews and randomised clinical trials, quasi-randomised trials or longitudinal cohort studies since the source guidelines published was conducted. The relevant publications were critically appraised and graded. The publications searched were between 2004 and June 2012. Year 2004 was the end date of search in the oldest source guideline by SIGN [18]. This limited search period was established to identify new publications that were not included in the three ‘source’ guidelines. The search was later updated to the period between 2004 and December 2012 following external review for the preparation of the final guidelines.

Draft-adapted/adopted recommendations and summaries of the evidence were assessed by the guideline development group for (1) consistency between the evidence presented, (2) its interpretation and the resulting adapted/adopted recommendations, and (3) the effect of any new evidence on the recommendations. For each of the draft-adapted/adopted recommendations, their acceptability and applicability in the local Hong Kong context, taking culture, organisation and expertise of dental services and population characteristic, beliefs and values into account, were discussed and considered in the meetings among the guideline development group. Using informal consensus, the recommendations were either adapted, updated or rejected based on the latest evidence identified. A preliminary list of 91 draft recommendations was developed.

### Finalisation phase

A consultation draft of the adapted guidelines was produced with the preliminary list of 91 draft recommendations. The draft was circulated among the guideline development group members for comment and was revised where necessary.

#### External review process

All 68 ordinary and associate members (excluding guideline development group members and panel members) of the HKSPD (key stakeholder) were invited to be external reviewers by email. Ordinary HKSPD members were either registered paediatric dentists or practitioners who had completed structured training in the specialty and were experts in Paediatric Dentistry in Hong Kong. Associate members of HKSPD were general medical or dental practitioners who were interested in the practice of Paediatric Dentistry. All of them would be the potential users of the developing guidelines.

Electronic copies of the consultation draft of the adapted guidelines were circulated to the external reviewers. The external reviewers were invited to contribute any comments on the consultation draft of the developing guidelines. An Internet-based structured questionnaire on a 5-point Likert scale was used to ask the external reviewers about their view on the strengths and weaknesses of the consultation draft, any need for modification, whether they would approve the draft guidelines and use them in their practice, and how it would impact or change their current practice. The 22 items about the external reviewers’ general view on the guidelines were added up to create a general view score ranging from 0 to 110, with a higher score indicating a more positive attitude towards the guidelines. The external reviewers consented to participate in the guideline external review process and for the data to be published.

#### Ranking of recommendations

A two-round Internet-based password-protected Delphi consensus process was conducted among the external reviewers. The Delphi process was carried out to collect opinions or comments from external reviewers so as to establish consensus recommendations for the guidelines. Before each round, external reviewers were reminded of the upcoming surveys by at least four emails. The draft recommendations were presented to the external reviewers. They rated their agreement with each of the 91 preliminary adapted/adopted recommendations using a 9-point Likert scale (1 = strongly disagree, 5 = no opinion or not enough information or experience to judge, 9 = strongly agree) and to contribute any comments on the recommendations. They were given the option of not commenting on a recommendation when the topic was outside their respective areas of expertise. This process was repeated during the second round, in which reviewers’ ratings, dissenting opinions or uncertainty, or absence of evidence were combined and provided anonymously to all of them. They were allowed to revise their first round responses in view of further responses in the second round. After the first round, the guideline development group met to modify some recommendations for clarity and presentation for the second round.

#### Classification of recommendations

For the first round of Delphi, recommendations rated by < 20% of the external reviewers were discarded. Recommendations rated by 80% of the external reviewers were discarded if the overall median score fell within the bottom tertile (1–3, disagree) and deferred until the second round if the overall median score was 4–6 (neutral). Recommendations with overall median scores within the top tertile (7–9) were classified as follows: those rated by < 80% of the external reviewers were deferred until the second round, and those rated by ≥ 80% of the external reviewers were classified as ‘high agreement’ if they had an overall median score of 9 or as ‘agreement’ they had an overall median score of 7 or 8.

**Fig. 1:**
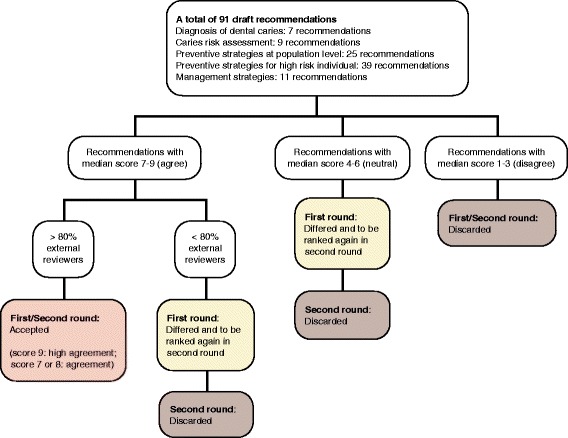
Classification of recommendations according to the overall median score and percentage of ratings by external reviewers in the first and second round of Delphi surveys

Similarly, in the second round of Delphi, recommendations rated by < 20% of the external reviewers were discarded. Recommendations rated by 80% of the external reviewers were discarded if the overall median score fell between 1 and 6. Recommendations with an overall median score within the top tertile (7–9) were classified as follows: those rated by < 80% of the external reviewers were discarded, and those rated by ≥ 80% of the external reviewers were classified as for the first round. The classification of recommendations according to the overall median score and percentage of ratings by external reviewers in the two-round Delphi process is illustrated in Fig. 1.

#### Data analysis

Data were analysed using IBM® SPSS Statistics 19 (SPSS Inc., Chicago, IL, USA). Recommendation-specific medians were estimated for each round. The inter-rater agreement between participants in each round was evaluated using intraclass correlation coefficient with 95% confidence intervals (CI). Microsoft Excel for Mac 2011 was used for preparing tabular and graphic presentation.

#### Production of the final guideline

All the feedback from the external reviewers were documented and presented in the guideline development group meeting for discussion. The guidelines and recommendations were further revised to incorporate feedback from the external reviewers where necessary.

## Results

**Fig. 2:**
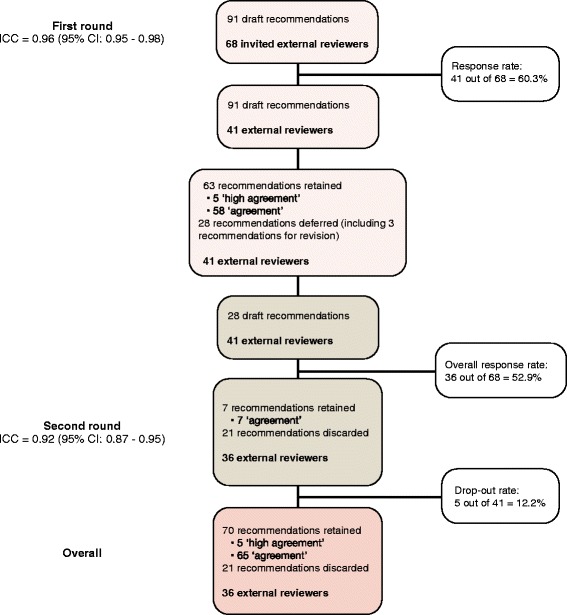
Recommendations and external reviewers’ participation flow chart

The flow chart in Fig. 2 describes the number of guideline recommendations and participation of external reviewers in the external review process. Forty-one out of 68 invited members agreed to participate (response rate: 41/68 = 60.3%). In the Delphi process, there were 41 external reviewers in the first round and 36 for the second round (drop-out rate: 5/41 = 12.2%; overall response rate: 36/68 = 52.9%). The intraclass correlation coefficients were 0.96 (95% CI, 0.95–0.98) and 0.92 (95% CI, 0.87–0.95) for the first and second rounds, respectively.

All the participated external reviewers rated all the items. Of the original 91 recommendations, 70 were retained and classified as follows: five items reached consensus (‘high agreement’), 65 items reached near consensus (‘agreement’), 21 items were discarded. Results of the recommendations in the two-round Internet-based Delphi consensus surveys are listed in Table 2. Only those recommendations (*N* = 70) that had gained consensus from the external reviewers in the Delphi process were included in the final list of recommendations of the guidelines.

**Table 2:** Results of the recommendations of the adapted guidelines in the two-round Delphi survey

Topic	No.	Recommendations	First round^a^ Median Score (%)	Second round^a^ Median Score (%)	Final decision
Diagnosis of dental caries (*N* = 7)	1	Caries should be diagnosed as early as possible to allow management before cavitation and pulpal involvement, and to identify caries-active patients and those at increased risk of caries in the future	8, 100%	–	retained as ‘agreement’
	2	New caries detection systems, like Diagnodent, DIFOTI and QLF, can be used as an adjunct diagnostic method in addition to the traditional clinical and radiographic examination	7, 53.7%	7, 52.8%	discarded
	3	A thorough clinical examination should be carried out on clean dried teeth	8, 87.8%	–	retained as ‘agreement’
	4	As thorough a caries diagnostic examination should be performed as the child’s level of cooperation permits	8, 87.8%	–	retained as ‘agreement’
	5	The use of bitewing radiography for caries diagnosis should be considered for pre-school children attending for dental care, particularly if they are assessed as being at increased risk of dental caries	6, 48.8%	6.5, 50.0%	discarded
	6	The timing of subsequent radiographic examinations should be based on the patient’s caries risk status	8, 90.2%	–	retained as ‘agreement’
	7	Practitioners should receive training in clinical and radiographic caries diagnosis	8, 87.8%	–	retained as ‘agreement’
Caries risk assessment (*N* = 9)	8	The use of a practical caries risk assessment tool should be implemented into practice to facilitate the caries risk assessment process	7, 68.3%	8, 86.1%	retained as ‘agreement’
	9	The caries risk assessment tool should be integrated into the electronic patient record	7, 58.5%	8, 75.0%	discarded
	10	Public health nurses, practice nurses, general practitioners and other primary care workers who have regular contact with young children should have training in the identification of high caries risk pre-school children	8, 80.5%	–	retained as ‘agreement’
	11	Public health nurses or other child healthcare professionals should carry out a caries risk assessment for children as part of their routine overall health assessment and recorded in the child’s health record	7, 73.2%	8, 83.3%	retained as ‘agreement’
	12	Referral pathways should be developed to allow referral of high caries risk pre-school children from primary, secondary and social care services into dental services	8, 82.9%	–	retained as ‘agreement’
	13	A dental practice-based caries risk assessment should be carried out on individual pre-school children and should include the following risk indicators: – evidence of previous caries experience – resident in a deprived area – healthcare worker’s opinion – oral mutans streptococci counts (if accessible)	7, 61.0%	–	
	13	*Revised in second round to:* A dental practice-based caries risk assessment should be carried out on individual pre-school children and should include the following risk indicators: – evidence of previous caries experience – medically compromised or with special needs – lower socioeconomic family background – resident in a deprived area – healthcare worker’s opinion – oral mutans streptococci counts (if accessible) *(revised as some external reviewers suggested that more caries risk factors should be included)*	–	8, 80.6%	retained as ‘agreement’
	14	A formal caries risk assessment using a structured caries risk assessment tool should be done for children attending the dental clinic for dental assessment or emergency care	8, 68.3%	8, 80.6%	retained as ‘agreement’
	15	Recall of children for re-assessment of caries risk should be based on the clinician’s assessment of the child’s caries risk status using the caries risk assessment tool, and should not exceed 12 months	8, 78.0%	8, 88.9%	retained as ‘agreement’
	16	Children whose families live in a deprived area should be considered as at increased risk of early childhood caries when developing preventive programs	7, 70.7%	–	
		*Revised in second round to:* Children whose families live in a deprived area or from lower socioeconomic background should be considered as at increased risk of early childhood caries when developing preventive programs *(revised as some external reviewers suggested a more detailed socioeconomic background should be assessed, rather than just the residential area of patient)*	–	8, 77.8%	discarded
Preventive strategies for pre-school children at population level (*N* = 25)	*Oral health education*				
	17	Oral health education and dietary advice should be incorporated into each child’s general well-being developmental check or at any appropriate opportunity that arises	8, 97.6%	–	retained as ‘agreement’
	18	The oral health of young children should be promoted through multiple interventions and multi-sessional health promotion programmes for parents, and if possible, incorporated into relevant general health promotion interventions	8, 97.6%	–	retained as ‘agreement’
	19	The dental health team should deliver caries prevention strategies in conjunction with physical prevention techniques such as oral hygiene instruction and the use of fluoride	8, 100%	–	retained as ‘agreement’
	20	Oral health promotion programmes for young children should be initiated before the age of 3 years	8, 95.1%	–	retained as ‘agreement’
	21	Parents should be encouraged to take their children for regular dental care as soon as the first teeth erupt	8, 90.2%	–	retained as ‘agreement’
	22	Teachers, community workers and lay or peer educators can be effective in delivering health promotion interventions and their role should be considered in the development of oral health promotion programmes	8, 82.9%	–	retained as ‘agreement’
	23	Non-dental health professionals and lay oral health workers should be provided with adequate educational or training interventions prior to their participation in oral health promotion programmes	8, 90.2%	–	retained as ‘agreement’
	24	Multidisciplinary approaches across a range of settings should be taken in the delivery of oral health promotion programmes	8, 87.8%	–	retained as ‘agreement’
	25	The use of consistent oral health messages should be promoted to support multidisciplinary approaches within oral health promotion programmes	8, 90.2%	–	retained as ‘agreement’
	26	Oral health promotion programmes to reduce the risk of early childhood caries should be available for parents during pregnancy and continued postnatally	8, 97.6%	–	retained as ‘agreement’
	*Dietary advice*				
	27	The use of xylitol by parents or carers to reduce caries in their children should be considered	6, 39.0%	6, 36.1%	discarded
	28	Oral health education on diet to parents/carers and children should start early in life, encouraging balanced healthy eating and a reduction in both frequency and total amount of sugars ingested	8, 100%	–	retained as ‘agreement’
	*Use of fluoride*				
	29	Community or home-based oral health promotion interventions should use fluoride-containing agents such as fluoride toothpaste	9, 95.1%	–	retained as ‘high agreement’
	30	Community-based tooth-brushing programmes should include fluoride toothpaste with a concentration of 1000 ppm F	8, 80.5%	–	retained as ‘agreement’
	31	Community-based tooth-brushing programmes should be undertaken in community-based settings such as nurseries	8, 87.8%	–	retained as ‘agreement’
	32	Community-based tooth-brushing programmes should be undertaken with parents to create a supportive environment for oral health behaviour	8, 97.6%	–	retained as ‘agreement’
	*Tooth-brushing*				
	33	Parents/carers should brush their child’s teeth as soon as the first tooth appears	9, 95.1%	–	retained as ‘high agreement’
	34	All children should be encouraged to brush their teeth under adult supervision	8, 95.1%	–	retained as ‘agreement’
	35	All children should be encouraged to brush their teeth under adult supervision with fluoride toothpaste containing at least 1000 ppm F	8, 70.7%	8, 75.0%	discarded
	36	All children should be encouraged to brush their teeth under adult supervision twice a day	8, 85.4%	–	retained as ‘agreement’
	37	All children should be encouraged to brush their teeth under adult supervision at bedtime and one other time during the day	8, 80.5%	–	retained as ‘agreement’
	38	All children should be encouraged to brush their teeth under adult supervision using a smear (age < 2) or small pea size (age 2–7) amount of toothpaste	8, 82.9%	–	retained as ‘agreement’
	39	Children should be encouraged to spit out toothpaste and not rinse after brushing	8, 70.7%	8, 69.4%	discarded
	40	Pre-school children should be supervised by an adult when brushing their teeth	8, 100%	–	retained as ‘agreement’
	41	Preventive programmes comprising combinations of interventions that include fluoride or fissure sealants should be considered for children based on their caries risk status	8, 100%	–	retained as ‘agreement’
Preventive strategies for pre-school children at high risk (*N* = 39)	*Caries risk assessment*				
	42	A formal caries risk assessment using a structured caries risk assessment tool should be done for children attending the dental clinic for dental assessment or emergency care, and should be re-assessed at certain interval	8, 82.9%	–	retained as ‘agreement’
	43	Recall of children for re-assessment of caries risk should be based on the clinician’s assessment of the child’s caries risk status using the caries risk assessment tool, and should not exceed 12 months	8, 82.9%	–	retained as ‘agreement’
	*Diet*				
	44	Oral health education on diet to parents/carers and children should start early in life, encouraging balanced healthy eating and a reduction in both frequency and total amount of sugars ingested	8, 97.6%	–	retained as ‘agreement’
	45	Parents/carers should be encouraged to limit their child’s consumption of sugar-containing foods and drinks, and when possible, to confine their consumption to mealtimes	9, 100%	–	retained as ‘high agreement’
	46	Children should be encouraged to limit their consumption of sugar-containing foods and drinks, and when possible, to confine their consumption to mealtimes	8, 100%	–	retained as ‘agreement’
	47	Parents and carers of children should be advised that drinks containing free sugars, including natural fruit juices, should be avoided between meals, and should never be put into a feeding bottle. Water may be given instead	8, 95.1%	–	retained as ‘agreement’
	48	Parents and carers should be advised not to let their child sleep or nap with a baby bottle or feeder cup	9, 100%	–	retained as ‘high agreement’
	49	Members of the dental team should support and promote breastfeeding according to current WHO recommendations (infants should be breast fed for first 6 months, and after should receive complementary foods with continued breastfeeding up to 2 years or beyond)	8, 78.0%	8, 72.2%	discarded
	50	Parents and carers should be advised that soya infant formulae are potentially cariogenic and should be used only when medically indicated	7, 70.7%	8, 72.2%	discarded
	51	Sugar-free formulations of medicines should be used if available and if not parents and carers should be advised to give doses with meals and never after tooth-brushing at night	8, 82.9%	–	retained as ‘agreement’
	52	Parents and carers should be advised that cheese is a good high energy food for toddlers as it is non-cariogenic and may be actively protective against caries	8, 85.4%	–	retained as ‘agreement’
	53	Parents and carers should be assured that sugar-free snacks are unlikely to be cariogenic	7, 65.9%	7, 63.9%	discarded
	54	Parents and carers should be advised that confectionery and beverages containing sugar substitutes are preferable, but should be consumed in moderation	7, 68.3%	8, 61.1%	discarded
	*Topical fluoride*				
	55	The use of fluoride mouthrinse is not recommended for pre-school children due to the risk of fluoride ingestion	8, 95.1%	–	retained as ‘agreement’
	56	Topical fluoride varnish application (22,600 ppm F) should be given to pre-school children who are assessed as being at increased caries risk, at intervals of every 3 or 6 months	8, 92.7%	–	retained as ‘agreement’
	57	Fluoride varnish should be used in preference to fluoride gel for caries prevention in children who are assessed as being at high caries risk	8, 80.5%	–	retained as ‘agreement’
	58	Fluoride gel should not be used in children under the age of 7	7, 53.7%	6, 47.2%	discarded
	*Toothbrush & Toothpaste*				
	59	Children should have their teeth brushed with fluoride toothpaste	8, 95.1%	–	retained as ‘agreement’
	60	Parents/carers should be brush their child’s teeth as soon as the first tooth appears	8, 92.7%	–	retained as ‘agreement’
	61	Toothpaste containing 1000 ppm F ±10% should be used by pre-school children	7, 68.3%	7, 58.3%	discarded
	62	Children should have their teeth brushed, or be supervised/assisted with tooth-brushing by an adult, at least twice a day, with a smear or pea-sized amount of fluoride toothpaste	8, 92.7%	–	retained as ‘agreement’
	63	Children’s teeth should be brushed last thing at night, before bedtime and on at least one other occasion	8, 82.9%	–	retained as ‘agreement’
	64	Children should be encouraged to spit out excess toothpaste and not rinse with water post-brushing	7, 70.7%	7.5, 63.9%	discarded
	65	Eating directly after brushing should be avoided to prevent fluoride from being washed out of the mouth prematurely	7, 51.2%	7, 55.6%	discarded
	66	Brushing children’s teeth with powered toothbrush with a rotation oscillation can be more effective	6, 34.1%	6.5, 50.0%	discarded
	67	Parents and carers should use a toothbrush with a small head for children	8, 92.7%	–	retained as ‘agreement’
	68	For pre-school children age < 2, parents/carers of children should be encouraged to brush their child’s teeth with fluoride toothpaste containing at least 1000 ppm F	5, 34.1%	4, 25.0%	discarded
	69	For pre-school children age < 2, parents/carers of children should be encouraged to brush their child’s teeth twice a day	8, 90.2%	–	retained as ‘agreement’
	70	For pre-school children age < 2, parents/carers of children should be encouraged to brush their child’s teeth at bedtime and one other time during the day	8, 82.9%	–	retained as ‘agreement’
	71	For pre-school children age < 2, parents/carers of children should be encouraged to brush their child’s teeth using a smear of toothpaste	8, 82.9%	–	retained as ‘agreement’
	72	For pre-school children age > 2, all children should be encouraged to brush their teeth under adult supervision	8, 100%	–	retained as ‘agreement’
	73	For pre-school children age > 2, all children should be encouraged to brush their teeth under adult supervision with fluoride toothpaste containing at least 1000 ppm F	8, 63.4%	8, 72.2%	discarded
	74	For pre-school children age > 2, all children should be encouraged to brush their teeth under adult supervision twice a day	8, 90.2%	–	retained as ‘agreement’
	75	For pre-school children age > 2, all children should be encouraged to brush their teeth under adult supervision at bedtime and one other time during the day	8, 82.9%	–	retained as ‘agreement’
	76	For pre-school children age > 2, all children should be encouraged to brush their teeth under adult supervision using a small pea size amount of toothpaste	8, 82.9%	–	retained as ‘agreement’
	*Fissure sealants*				
	77	Children who are assessed as being at high caries risk should have resin-based fissure sealant applied and maintained in vulnerable pits and fissures of permanent teeth	8, 97.6%	–	retained as ‘agreement’
	78	Routine application of sealants on primary molar teeth is not recommended, but may be considered for selected high caries risk children	8, 85.4%	–	retained as ‘agreement’
	*Anti-microbial agent*				
	79	The use of chlorhexidine for caries prevention is not recommended	8, 68.3%	8, 75%	discarded
	*Re-mineralising product*				
	80	The use of re-mineralising products (e.g. CPP-ACP) can help caries prevention	8, 78%	8, 88.9%	retained as ‘agreement’
Management strategies (*N* = 11)	81	Primary teeth with caries progressing into dentine should be actively managed with a preventive, or a preventive and restorative, approach as appropriate with the child’s ability to cooperate	8, 90.2%	–	retained as ‘agreement’
	82	Restorative treatment should always be provided in conjunction with a course of preventive treatment	8, 90.2%	–	retained as ‘agreement’
	83	If complete caries removal from a vital primary molar is not possible an indirect pulp capping technique should be considered	8, 87.8%	–	retained as ‘agreement’
	84	A calcium hydroxide containing lining material, followed by an adhesive restoration or a pre-formed metal crown, should be used	7, 65.9%	6, 47.2%	discarded
	85	When preparing a Class II cavity, care must be taken to avoid iatrogenic damage to adjacent proximal tooth surfaces	9, 100%	–	retained as ‘high agreement’
	86	Use of the Atraumatic Restorative Treatment approach for cavity preparation in carious primary teeth should be considered as an alternative, where appropriate, to conventional cavity preparation techniques	8, 90.2%	–	retained as ‘agreement’
	87	Minimal amount of formocresol for doing pulpotomy in primary teeth should be used	8, 80.5%	–	retained as ‘agreement’
	88	Amalgam, composite, resin-modified glass-ionomers, compomer or preformed metal crowns should be used as restorative materials for cavities in primary molars; conventional glass-ionomer should be avoided, where possible, for Class II cavity restoration	8, 95.1%	–	
	88	*Revised in second round to:* Composite, resin-modified glass-ionomers, compomer or preformed metal crowns should be used as restorative materials for cavities in primary molars; conventional glass-ionomer should be avoided, where possible, for Class II cavity restoration *(revised as some external reviewers commented that amalgam should better not to be used in children)*	–	8, 86.1%	retained as ‘agreement’
	89	Conventional glass-ionomer should be avoided, where possible, for Class II cavity restoration	7, 56.1%	8, 63.9%	discarded
	90	Tooth bonding adhesives allows more conservative preparation and should be used according to manufacturer’s instruction	8, 95.1%	–	retained as ‘agreement’
	91	Clinical management protocols, based on a child’s age, caries risk, and level of patient/parent cooperation, provide health providers with criteria and protocols for determining the types and frequency of diagnostic, preventive, and restorative care for patient specific management of dental caries	8, 92.7%	–	retained as ‘agreement’

^a^Numbers are median score; ratings percentages by external reviewers with an overall median score within the top tertile (7–9)

The ratings of the external reviewers about their general views on the strengths and weaknesses of the guidelines are listed in Table 3. The majority of the external reviewers (82.9%) agreed that there was a need for the guidelines on caries prevention and management by caries risk assessment for pre-school children in Hong Kong (statement 2). Over half of them (>60%) agreed to practice the guidelines when they are implemented (statement 21–22) and agreed that the guidelines were properly developed (statement 1, 3, 4). They were also positive about the relevance and applicability of guideline recommendations in practice and on patients (statement 5–13, 19). However, their views regarding the possible effects and outcomes of the guidelines were neutral (statement 14–18). Over 60% of the external reviewers approved the guidelines as clinical practice guidelines. The mean general view score of the guideline was 75.66 (SD = 7.80; out of a maximum possible score of 110).

**Table 3:** General view of the external reviewers on the strengths and weaknesses of the guideline

No.	Statement about the view on the guideline	Median score	Percentage of score at 4–5
1	The rationale for developing a guideline, as stated in the ‘1. Introduction’ section of the draft guideline report, is clear	4	73.2
2	There is a need for a guideline of this topic	4	82.9
3	The literature search is relevant and complete in this draft guideline (i.e. no key trials were missed nor any included that should not have been)	4	61
4	I agree with the methodology used to summarize the evidence included in this draft guideline	4	63.4
5	The draft recommendations in this report are clear	4	73.2
6	I agree with the draft recommendations as stated	4	68.3
7	The draft recommendations are suitable for the pre-school children	4	70.7
8	The draft recommendations are too rigid to apply to individual patients	3	36.6
9	When applied, the draft recommendations will produce more benefits for patients than harms	4	90.2
10	The draft guideline report presents options that will be acceptable to patients	4	68.3
11	To apply the draft recommendations will require reorganization of services/care in my practice setting	4	58.5
12	To apply the draft recommendations will be technically challenging	3	26.8
13	The draft recommendations are too expensive to apply	3	31.7
14	The draft recommendations are likely to be supported by a majority of my colleagues	3	34.1
15	If I follow the draft recommendations, the expected effects on patient outcomes will be obvious	3	39
16	The draft recommendations are the same as the current usual practice	3	43.9
17	The draft recommendations reflect a more effective approach for improving patient outcomes than is current usual practice	3	41.5
18	When applied, the draft recommendations will result in better use of resources than current usual practice	3	26.8
19	I would feel comfortable if my patients received the care recommended in the draft guideline	4	70.7
20	The draft guideline should be approved as a practice guideline	4	61
21	If this draft guideline were to be approved, I would make use of it in my own practice	4	63.4
22	If this draft guideline were to be approved, I would apply the recommendations to my practice	4	68.3

Statements were rated on a 5-point Likert scale (1 = strongly disagree; 2 = disagree; 3 = neutral; 4 = agree; 5 = strongly agree)

## Discussion

Clinical practice guidelines are the major means to summarise and translate rapidly changing research evidence into practice and assist clinical decision making [25, 26]. Clinicians require knowledge of the current expert recommendations and guidelines to manage specific clinical problems and improve their patients’ outcomes. Developing evidence-based guidelines on caries prevention and management by caries risk assessment for pre-school children in Hong Kong is important. The guidelines have great potential in improving the current oral health and dental care of pre-school children in Hong Kong if implemented.

Developing clinical practice guidelines using the ADAPTE process and Delphi consensus has not been reported in the literature. This approach allows developing guidelines with consensus evidence-based recommendations. The literature suggests that guidelines developed without active participation and involvement of the intended users and which were partially without credible scientific basis were not fully supported by the profession and were poorly accepted by the practitioners [27, 28]. ADAPTE is a comprehensive systematic framework for adapting guidelines produced in one setting to be used in a different cultural context. The framework was developed to meet the current challenges of guideline development and implementation that require lots of time, expertise and resources [29]. The process is designed for a range of stakeholders, such as guideline developers, healthcare providers, consumers and policymakers at the local, national and international levels and in different healthcare sectors [10]. The process also allows users to customise the process to their own context, resource constraints and expertise. In this project, in addition to the external review process set up in the ADAPTE framework, the Delphi consensus technique was employed to gain reviewers’ comments and consensus on the guideline recommendations. The developed guidelines are evidence-based with up-to-date knowledge and have gained consensus among the profession. Concerns in the acceptability and barriers in implementation are reduced.

A consensus approach using the Delphi technique was employed during the external review process of the guidelines. Delphi strengthened the external review process of this guideline development. In addition to external reviewers’ comments and views on the draft-adapted guidelines, their consensus on the guidelines’ recommendations was achieved. External reviewers were members of the *Hong Kong Society of Paediatric Dentistry*, who were either specialists of paediatric dentistry or dental/medical practitioners with active participation in the field. This heterogeneous sample ensured that a wide spectrum of opinions of the guideline could be collected. The group consisted of 41 members in round 1 and 36 members in round 2, thus reducing the potential of selection bias of ‘experts’ for the Delphi process [13, 14, 30]. Moreover, the invited external reviewers are likely to be the major users of the guidelines when implemented. Involving them in the external review process using Delphi promotes ownership and acceptance of the guidelines, ultimately reducing the barrier of guideline adherence in practice in the future [31]. Involvement of the general public in the external review process could be considered in the future. They are one of the key stakeholders of the guidelines. Their input could help develop more comprehensive evidence-based guidelines with minimum barriers during implementation.

The response rate in the second round of Delphi (52.9%, 36/68) dropped when compared to the first round (60.3%, 41/68). Recruitment of dental practitioners in a research study is a challenge and it is also difficult to retain a high response rate in each round of Delphi [13, 14]. Instead of employing the classical four-round Delphi, a two-round web-based Delphi was used [14]; the use of Internet avoids the need for face-to-face meetings among the external reviewers. The external reviewers can review the guidelines at different geographic locations and time.

The consensus level of the Delphi technique in the literature ranges from 51% to 80%; a bench mark of 70% is used in many cases [30]. We used 80% as the level of consensus in this guideline development project. The intraclass correlation and CIs were good. Consensus was reached for a large proportion of recommendations with high inter-rater agreement between rounds.

The online implementation of the Delphi technique for external review was well received. External reviewers completed the two-round web-based Delphi external review of the guideline over a period of 5 months. They contributed extensive and supportive comments on the guidelines and the recommendations.

Not all evidence-based recommendations were accepted by the external reviewers. Although the recommendation, ‘All children should be encouraged to brush their teeth under adult supervision with fluoride toothpaste containing at least 1000 ppm F’ was based on strong evidence [32], it was not able to gain agreement from the external reviewers. A number of external reviewers were concerned about dental fluorosis in relation to the use of high concentration fluoride toothpaste in a water fluoridated area like Hong Kong. This reflected that the strength of the evidence, applicability to real-life conditions and associated socioeconomic costs influenced the acceptance of recommendations. Moreover, this showed that the guideline development process was effective in identifying potential local barriers in applying research evidence to clinical practice and recommendations allowed their modification to fit local circumstances in response to reviewers’ opinions.

## Conclusions

Using the ADAPTE process and Delphi consensus, evidence-based clinical guidelines for caries prevention and management by caries risk assessment for pre-school children in Hong Kong were developed. These guidelines can facilitate the translation of evidence into practice. The framework for developing the guidelines could be employed elsewhere.

## Abbreviations

AGREE II, Appraisal of Guidelines for Research and Evaluation II instrument; HKSPD, Hong Kong Society of Paediatric Dentistry; SIGN, Scottish Intercollegiate Guideline Network (SIGN)
